# Learning to predict: Exposure to temporal sequences facilitates prediction of future events

**DOI:** 10.1016/j.visres.2013.10.017

**Published:** 2014-06

**Authors:** Rosalind Baker, Matthew Dexter, Tom E. Hardwicke, Aimee Goldstone, Zoe Kourtzi

**Affiliations:** a School of Psychology, University of Birmingham, Birmingham B15 2TT, UK; b Department of Psychology, University College London, London, UK; c Department of Psychology, University of Cambridge, Cambridge, UK

**Keywords:** Visual learning, Transfer, Perception, Prediction, Attention

## Abstract

Previous experience is thought to facilitate our ability to extract spatial and temporal regularities from cluttered scenes. However, little is known about how we may use this knowledge to predict future events. Here we test whether exposure to temporal sequences facilitates the visual recognition of upcoming stimuli. We presented observers with a sequence of leftwards and rightwards oriented gratings that was interrupted by a test stimulus. Observers were asked to indicate whether the orientation of the test stimulus matched their expectation based on the preceding sequence. Our results demonstrate that exposure to temporal sequences without feedback facilitates our ability to predict an upcoming stimulus. In particular, observers’ performance improved following exposure to structured but not random sequences. Improved performance lasted for a prolonged period and generalized to untrained stimulus orientations rather than sequences of different global structure, suggesting that observers acquire knowledge of the sequence structure rather than its items. Further, this learning was compromised when observers performed a dual task resulting in increased attentional load. These findings suggest that exposure to temporal regularities in a scene allows us to accumulate knowledge about its global structure and predict future events.

## Highlights

• Exposure to temporal sequences improves prediction of future events.
• Learning to predict from temporal sequences generalizes to untrained stimuli.
• Learning to predict is sensitive to the global structure of the trained sequence.
• Learning to predict is compromised by increased attentional load.

## 1 Introduction

Successful everyday interactions entail that we extract information about the structure of the environment and use it to recognize the current scene as well as predict future events. There is accumulating evidence that previous experience with the environment facilitates our ability to extract information about spatial and temporal regularities in cluttered scenes (Aslin and Newport, 2012, Perruchet and Pacton, 2006). This learning by mere exposure to stimuli that co-occur is known as statistical learning and has been shown to facilitate performance in a range of tasks: object recognition (Brady and Chun, 2007, Brady and Oliva, 2008), language understanding (Misyak, Christiansen, & Tomblin, 2010), social judgments (Kunda & Nisbett, 1986) and inductive reasoning (Kemp & Tenenbaum, 2009). For example, after exposure to items (e.g. shapes, tones or syllables) that co-occur in space or appear in a temporal sequence, observers report that structured combinations are more familiar than random contingencies (Chun, 2000, Fiser and Aslin, 2002a, Saffran et al., 1996, Saffran et al., 1999, Turk-Browne et al., 2005). Infants as young as 8-months old are able to parse meaningful linguistic units after exposure to a stream of syllables (Fiser and Aslin, 2002b, Saffran et al., 1996). Taken together, this previous work suggests that observers acquire implicit knowledge of the regularities present in a scene, despite the fact that they may not be explicitly aware of its specific structure. However, little is known about how we translate this knowledge of spatiotemporal structures to predictions of future events.

**Fig. 1:**
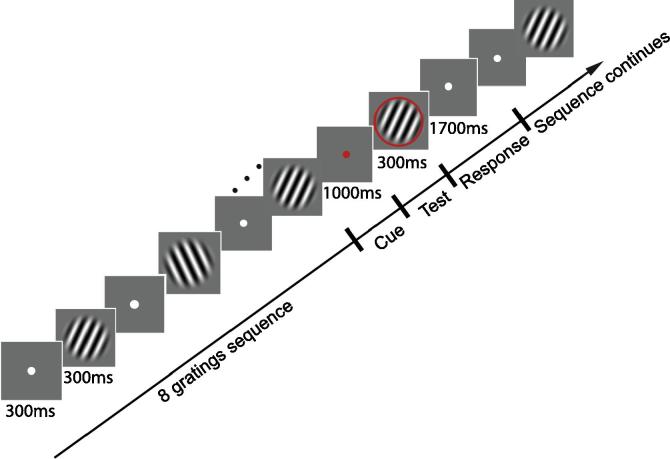
Stimuli and design: diagram illustrating the trial design: a sequence of eight gratings was repeated twice and was interrupted by the presentation of a cue and test stimulus. The sequence continued after the participants indicated their response until all eight gratings were presented indicating the end of the trial.

To address this question, here we test whether exposure to temporal sequences facilitates our ability to predict an upcoming stimulus. Previous studies have mainly used reaction times as an implicit measure of anticipation of an upcoming stimulus following implicit learning of temporal sequences (Nissen & Bullemer, 1987; for review see Schwarb & Schumacher, 2012). In contrast, here we ask whether learning a temporal sequence facilitates the explicit recognition of an upcoming stimulus. To this end, we asked participants to make an explicit prediction about the identity of the stimulus that they expected to appear following a temporal sequence. In particular, we presented observers with a sequence of leftwards and rightwards oriented gratings that was interrupted by a test stimulus (Fig. 1). Observers had to maintain attention throughout the temporal sequence as the temporal position of the test stimulus was randomly chosen across trials and were asked to indicate whether the test stimulus matched their expectation or not. Our results demonstrate that the observers’ ability to predict the orientation of the test stimulus improved following exposure to structured but not random sequences, suggesting that observers use information about temporal structure to predict future events. We further investigate whether learning to predict in the context of temporal sequences generalizes to new structures and stimuli and whether it is affected by attentional load.

## 2 Methods

### 2.1 Observers

Sixty-six healthy undergraduate students from the University of Birmingham took part in the study: (Experiment 1: *n* = 16 (5 male, 11 female; mean age = 21.25 years); Experiment 2: *n* = 18 (6 male, 12 female; mean age = 19.9 years); Experiment 3: *n* = 18 (12 male, 6 female; mean age = 20.9 years); Experiment 4: *n* = 14 (5 male, 9 female; mean age = 21.5 years). All participants were naïve to the aim of the study, had normal or corrected-to-normal vision, had no history of neurological disorders and gave written informed consent. This study was approved by the University of Birmingham Ethics Committee.

### 2.2 Stimuli

Stimuli comprised two Gabor gratings presented at 11.6° visual angle, 100% contrast, and spatial frequency of .48 cycles per degree. The gratings were oriented clockwise or counter clockwise from the vertical axis (±10°) with the exception of Experiment 3 (see details below). To control for local adaptation across stimuli we jittered the grating orientation randomly within ±2° across trials. Stimuli were generated in Matlab R2009a (version 7.8.0.347) using the Psychophysics toolbox (version 3.0.8) (Brainard, 1997, Pelli, 1997). Stimuli were displayed at a distance of 450 mm on a middle gray background in the center of a 1280 × 1024 pixel (0.3 mm × 0.3 mm per pixel; 85 Hz refresh rate) gamma-corrected ViewSonic P225f CRT monitor in a silent, dark, testing room.

We used these stimuli to generate four sequences, each comprising of 8 gratings presented at a different order across sequences, as shown below (1 refers to the leftwards oriented grating at −10° and 2 refers to the rightwards oriented grating at +10°):

Sequence A: 2 1 2 1 1 2 1 2.
Sequence B: 1 1 2 1 2 2 1 2.
Sequence C: 1 2 1 1 2 2 1 2.
Sequence D: 2 2 1 1 1 2 1 2.

Each grating orientation was presented four times in each sequence. Each sequence was repeated twice, resulting in 16 stimuli per trial. As all gratings were presented at the same rate, observers could not use duration to group elements or segment the sequences. Further, to ensure that observers did not perform the task simply by memorizing the first or last stimuli in the sequence, the orientation of the first stimulus was randomized in each trial and the last three stimuli in each sequence were always the same. Finally, as the frequency of occurrence was matched for the two grating orientations in the sequence, observers were required to learn the order of the elements in the sequence (i.e. temporal order statistics among pairs or triplets of oriented gratings).

### 2.3 Design

All participants completed an initial practice (10 min) to familiarize themselves with the task procedure and timing of the sequence. During this practice session participants were presented with simple sequences (1 2 1 2 1 2 1 2 or 2 1 2 1 2 1 2 1: repeated to make a 16 item sequence) and were given auditory error feedback.

Following this practice, participants completed a minimum of 3 and a maximum of 6 training sessions (until a criterion performance level of 80% was reached) without feedback on consecutive days. During each session participants completed four experimental runs. Each run comprised of 40 trials. Participants were presented with either sequences A and B (20 trials per sequence) or C and D (20 trials per sequence). The order in which the two sequences were presented was randomized across trials. Each run lasted approximately 10 min and participants were required to have a minimum 2-min break between each run. Following training, participants completed a transfer test session on untrained sequences (two runs). This session followed exactly the same procedure as the training sessions, but used the alternative pair of sequences (e.g. if participants were trained on sequences A and B they were tested on sequences C and D). This allowed us to assess transfer of learning from trained to untrained sequences. To test whether learning transfer was due to general practice with the task, participants also completed one run with random sequences (i.e. for each trial we generated a sequence comprising 16 gratings that were presented in random order) during this transfer session.

For each trial, participants were asked to respond to a test stimulus that appeared in each sequence. This test stimulus was one of the sequence gratings, highlighted by a red circle. Participants were required to report whether the test image had the same orientation (left vs. right) as the grating they expected to appear in that position in the sequence. The test stimulus appeared only in the second repeat of the sequence and its temporal position was randomized across trials. The test stimulus could appear in any position in the sequence except the last three positions; stimuli in these positions were the same across trials. For each run, 50% of the test stimuli were presented at the correct orientation for their position in the sequence. Participants performed this task without feedback across all training sessions and the transfer test.

#### 2.3.1 Experiment 1

Half of the participants in Experiment 1 were trained on sequences A and B, and tested on sequences C and D, while the rest of the participants were trained on sequences C and D, and tested on sequences A and B. Participants were presented with gratings oriented clockwise and counter clockwise of the vertical axis (±10°, with ±2° jitter). Each grating in the sequence was presented, at central fixation, for 300 ms followed by a blank interval (ISI) of 300 ms. During the ISI a white fixation dot was presented in the center of the display. A red dot cue was presented for 1000 ms before the test stimulus appeared. The test stimulus was then presented for 300 ms. Participants had a further 1700 ms to respond to the test stimulus. A white fixation dot was displayed for 300 ms following the participants’ response, after which the sequence continued until all items in the sequence were displayed. Participants responded using keys 1 and 2 on the numeric keypad to signal correct (i.e. the test stimulus orientation matched the expected orientation in the sequence) and incorrect respectively. The end of the trial was marked with a black ‘X’, which was displayed for 1000 ms before the start of the next trial.

#### 2.3.2 Experiment 2

All parameters for this experiment matched those of Experiment 1. Sequences A and B were used for training while C and D were used for the transfer test. Participants were presented with gratings oriented clockwise and counter clockwise of the vertical axis (±10°, with ±2° jitter). There was no test stimulus; instead observers were presented with a red dot for 2000 ms. A white fixation dot was displayed for a further 300 ms following the participants’ response, after which the sequence continued until all items in the sequence were displayed. Participants were instructed to respond which grating orientation they expected to see at that position in the sequence; 1 on the number pad for left and 2 for right orientation. As with Experiment 1, the end of the sequence was marked with a black ‘X’, which was displayed for 1000 ms. After this, the next trial began.

#### 2.3.3 Experiment 3

For this experiment, all parameters were similar to Experiment 1. Sequences A and B were used for training. However, half the participants were presented with gratings oriented clockwise and counter clockwise of the vertical axis (±10°, with ±2° jitter), while the rest of the participants were presented with gratings oriented around the horizontal axis (±10° with ±2° jitter). To test whether learning transfers to a sequence with the same temporal structure but comprising of different stimuli, we tested participants with the same sequences as those used for training but comprising of gratings presented at the untrained orientation. That is, participants that had trained on the vertical gratings were then tested on the horizontal gratings during the transfer session and vice versa.

#### 2.3.4 Experiment 4

For this experiment, all parameters were similar to Experiment 1. Sequences A and B were used for training while C and D for the transfer test. Participants were presented with gratings oriented clockwise and counter clockwise of the vertical axis (±10°, with ±2° jitter). However, during this experiment participants were instructed to perform a contrast detection task in addition to the prediction task, resulting in increased attentional load. During each trial, up to 3 of the gratings were presented at a lower contrast than the rest of the gratings in the sequence. Stimulus contrast (minimum and maximum contrast levels were set at 0.4 and 1 respectively) was controlled using a 3-down-1-up staircase method resulting in 79.4% performance level. As well as responding to a test grating in each sequence (as in Experiment 1) participants also had to count how many contrast changes they detected during the sequence. Once the sequence trial had ended a green cue was presented for a maximum of 2000 ms, during which the participants had to enter a number corresponding to the number of low contrast stimuli they had detected during the trial (0–3). After the participants responded, a white fixation dot was presented for 300 ms indicating the start of the next trial.

## 3 Results and discussion

### 3.1 Experiment 1

**Fig. 2:**
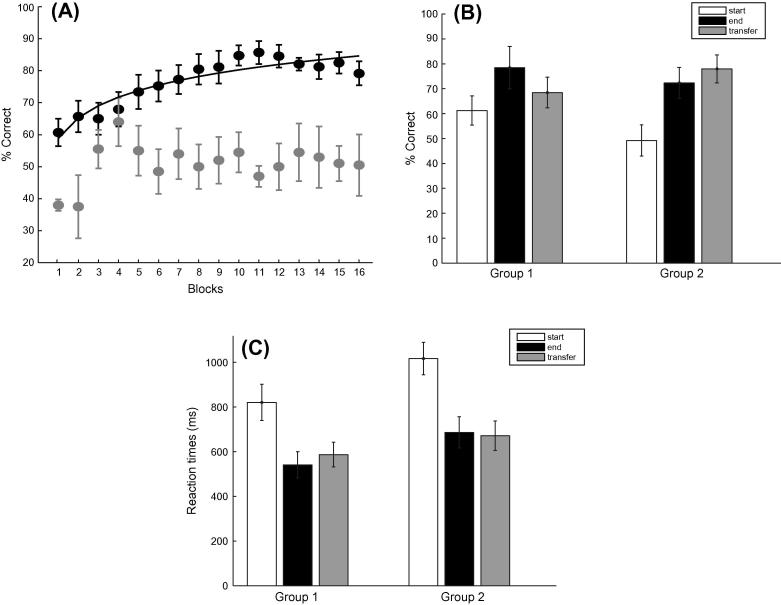
Experiment 1: (A) Behavioral performance (percent correct) across training blocks. Data is shown for strong learners (black circles; *n* = 11) and weak learners (gray circles; *n* = 5). Data is shown for 16 blocks (4 training sessions); some participants (*n* = 4) completed only three sessions, as their performance had already saturated above 80%; while one participant required additional sessions (*n* = 1 for 6 sessions). Data for strong learners are fitted (least squares non-linear fit) using the following equation: *y* = *k* * log(*x*) + *c*; where, *k* = the value of the tangent of the curve at *x* = 1, *c* = value of *y* for *x* = 1. (B) Percent correct performance is shown for the start (mean of first two training runs of the first session), end (mean of last two training runs of the last session) and transfer (mean of first two runs) blocks of the two sequences. Data is shown from two groups of participants (Group1: training with sequences A and B; Group2: training with sequences C and D). (C) Mean reaction times across all participants for the start, end and transfer sessions. Error bars indicate standard error of the mean.

Training enhanced the observers’ performance in predicting the correct test grating. As shown in Fig. 2A, most observers (11/16) showed increased performance across training blocks. For a small number (5/16) of weak learners (i.e. participants that reached performance less than 70% after a minimum of 4 training sessions) performance remained on average at 50.75% after training for four sessions. While performance improved for trained sequences, when observers were tested with random sequences performance remained on average at 55.78%, suggesting that this behavioral improvement reflects knowledge of the sequence structure rather than general familiarity with the task. To quantify this learning effect we compared the mean of the first two training blocks with the mean of the last two training blocks across all participants (including weak learners). A repeated measures ANOVA with Session (start, end of training), Group (Group1 was trained on sequences A, B, while Group 2 on sequences C, D) and Sequence type (A vs. B, or C vs. D) showed a main effect of Session (*F*(1, 14) = 26.34, *p* < 0.001) but no significant effect of Group (*F*(1, 14) < 1, *p* = 0.697) or Sequence Type (*F*(1, 14) < 1, *p* = 0.418). Further, we observed a significant interaction between Group and Session (*F*(1, 14) = 7.71, *p* = 0.01), consistent with better performance before training for observers trained on sequence A and B than observers trained on sequences C and D.

We then tested whether learning of the trained sequences generalized to untrained ones by comparing the mean of two transfer blocks to the start (mean of two first training blocks) and end (mean of last two training blocks) of training. Fig. 2B shows that generalization of learning dependent on the sequences used for training and test. A repeated measures ANOVA comparing the start and transfer of training with Session (start, transfer of training), Group (Group1 vs. Group 2) and Sequence type (A vs. B, or C vs. D) showed a significant interaction (*F*(1, 14) = 19.26, *p* < 0.01) between Session and Group. In particular, participants trained on sequences C and D showed better performance in the transfer test (i.e. untrained sequences) compared to the start of training (*t*(15) = −7.579, *p* > 0.001), while participants trained on sequences A and B did not show any significant difference between the transfer test and the start of training (*t*(15) = −0.157, *p* = 0.877). Similar analysis comparing the end of training (mean of last two training sessions) to the transfer test showed a complementary pattern of results. In particular, we observed a significant interaction (*F*(1, 14) = 5.61, *p* < 0.05) between Session and Group; that is, participants trained on sequences C and D showed similar performance in the transfer test compared to the end of training (*t*(15) = −0.928, *p* = 0.539), while participants trained on sequences A and B showed significant differences between the transfer test and the end of training (*t*(15) = 2.563, *p* < 0.05).

These results could be better understood when considering the observers’ performance for the two sets of sequences used in this experiment. In particular, sequences A and B were shown to be easier than sequences C and D, as indicated by better performance at the start of training for the former than the latter (*F*(1, 14) = 7.71, *p* = 0.01). This could be due to the fact that sequences A and B comprise 1 triplet (2 1 2) and 1 pair (1 1), while sequences C and D comprise three triplets (1 2 1, 2 1 2, 2 2 1) and two pairs (2 2, 1 1). Our results suggest that training generalized fully when participants were trained on a more difficult set of sequences but tested with an easier one. In contrast, when participants were trained on an easier set of sequences but tested with a more difficult one performance for the untrained sequences was similar to performance at the start of training with the easy sequence. In this case performance on the untrained sequence was better than at the start of training for the more difficult sequence (*t*(7) = −2.638, *p* < 0.05) suggesting potentially weaker transfer of learning to an untrained harder sequence. It is possible that when tested with easy sequences observers could identify item combinations (i.e. chunks) that were common between trained and untrained sequences (2 1 2, 1 1) resulting in transfer of learning to untrained sequences with a different global structure.

Analysis of reaction times (Fig. 2C) showed similar learning effects; that is observers’ responses became faster with training as supported by significant differences between sessions (start vs. end of training) (*F*(1, 14) = 53.307, *p* < 0.001). Transfer of this learning effect to untrained sequences depended on the difficulty of the untrained sequences. In particular, a repeated measures ANOVA on the data from all participants showed a significant interaction between Session (end vs. transfer of training) and Group (1 vs. 2) (*F*(1, 14) = 6.089, *p* < 0.05), consistent with the analysis on performance accuracy. Observers were shown to be faster when trained with a difficult sequence and tested with an easier untrained sequence.

**Fig. 3:**
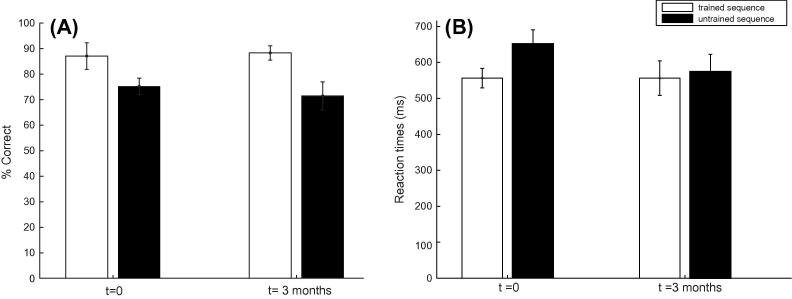
Experiment 1: (A) Behavioral performance (percent correct) is shown for strong learners (*n* = 7) during initial testing (*t* = 0) and three months later (*t* = 3 months). Data are shown for trained (mean of the last two runs of the final training session), and untrained (mean of two transfer runs) sequences. (B) Mean reaction times for trained and untrained sequences at *t* = 0 and *t* = 3 months. Error bars indicate standard error of the mean.

Further, we asked whether the learning effect we observed lasted over time. To this end, we tested six participants at a later time (3 months on average: range of 84–108 days) after their training on both the trained (A, B sequence) and untrained (C, D transfer) sequence. A repeated measures ANOVA showed a significant effect of Sequence (mean of last two runs of trained sequence vs. mean of two transfer blocks of untrained sequence) (*F*(1, 5) = 17.11, *p* < 0.01) but no significant effect of Session (original vs. delayed) (*F*(1, 5) = 1.47, *p* = 0.28), nor a significant interaction (*F*(1, 5) = 0.86, *p* < 0.781) between Session and Sequence, suggesting that observers’ performance remained the same three months after the initial training; that is, observers continued to perform better for trained than untrained sequences (Fig. 3). Similarly, analysis of reaction times (Fig. 3B) showed no significant effects of Sequence (*F*(1, 5) = 4.45, *p* = 0.08) or Session (*F*(1, 5) = 1.33, *p* = 0.30), nor a significant interaction (*F*(1, 5) = 4.68, *p* = 0.08) between Session and Sequence, suggesting that improvement in response speed due to learning did not change for a prolonged period after training.

### 3.2 Experiment 2

**Fig. 4:**
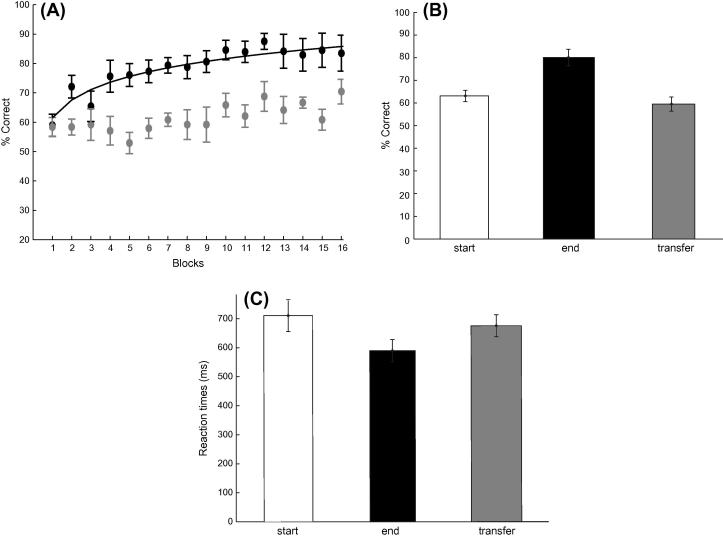
Experiment 2: (A) Behavioral performance (percent correct) across training blocks. Fitted data is shown for strong learners (black circles; *n* = 12) and weak learners (gray circles; *n* = 6). Data is shown for 16 blocks (4 training sessions); some participants (*n* = 5) completed only three sessions, as their performance had already saturated above 80%; while others required additional sessions (*n* = 4 for 5 sessions; *n* = 1 for 6 sessions). (B) Mean percent correct performance is shown across all participants for the start (mean of first two training runs of the first session), end (mean of last two training runs of the last session) and transfer (mean of first two runs) blocks. (C) Mean reaction times across all participants for the start, end and transfer sessions. Error bars indicate standard error of the mean.

To directly test whether the observers’ decision related to the prediction of the test stimulus, we tested performance in a new set of participants when the test stimulus was removed and observers were asked to explicitly predict the orientation (left vs. right) of the following grating in the sequence. All observers were trained on sequence A and B and tested on C and D. The results showed a similar pattern as in Experiment 1 (Fig. 4). Most observers (12/18) showed increased performance across training blocks, while for a small number (6/18) of weak learners (i.e. participants that reached performance less than 70% after a minimum of 4 training sessions) performance remained on average at 61.7% after training for four sessions. Observers’ performance improved during training, as shown by comparing performance at the start vs. the end of training, but remained at chance levels (mean performance of 48.53%) for random sequences. A repeated measures ANOVA on the data from all participants (including weak learners) with Session (start, end of training) and Sequence type (A vs. B, or C vs. D) showed a main effect of Session (*F*(1, 17) = 20.52, *p* < 0.01) but no significant effect of Sequence Type (*F*(1, 17) = 0.39, *p* = 0.539) nor a significant interaction between Session and Sequence Type (*F*(1, 17) = 0.21, *p* = 0.649).

We then tested whether learning of the trained sequences generalized to untrained ones. Comparing the mean of two transfer blocks to the start of training (mean of two first training blocks) showed no significant differences in performance (*F*(1, 17) = 1.05, *p* = 0.319). In contrast, comparing the mean of two transfer blocks to the end of training (mean of two last training blocks) showed significant differences in performance (*F*(1, 17) = 49.17, *p* < 0.001). Taken together these results suggest lack of significant generalization of learning to untrained sequences. Note that this result is consistent with Experiment 1, as observers were trained on the easier set of sequences and tested for transfer of learning on the harder set.

Analysis of reaction times (Fig. 4C) showed similar learning effects; that is, observers became faster with training. However when considering the data from all participants (both strong learners and weak learners) differences in reaction times were only marginally significant (*F*(1, 17) = 3.19, *p* = 0.092). This learning effect was specific to trained sequences and did not transfer to untrained ones. That is, there was no significant difference in reactions times between the start and transfer of training (*F*(1, 17) < 1, *p* = 0.903), but a marginally significant difference was observed between the end and transfer of training to an untrained sequence (*F*(1, 17) = 4.08, *p* = 0.059).

### 3.3 Experiment 3

**Fig. 5:**
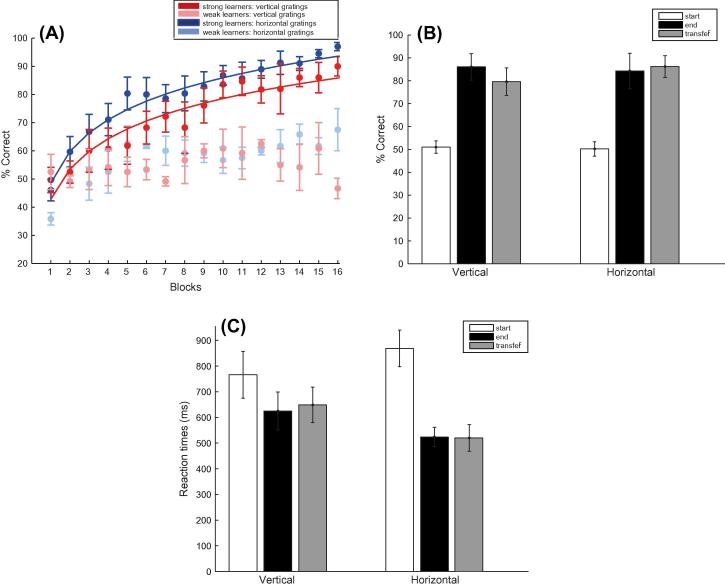
Experiment 3: (A) Behavioral performance (percent correct) across training blocks. Fitted data is shown for strong learners trained with vertical gratings (red circles; *n* = 7), strong learners trained with horizontal gratings (blue circles; *n* = 7), weak learners trained with vertical gratings (light red circles; *n* = 3) and weak learners trained with horizontal gratings (light blue circles; *n* = 3). Data is shown for 16 blocks (4 training sessions); some participants (*n* = 5) completed only three sessions, as their performance had already saturated above 80%; while others required additional sessions (*n* = 3 for 5 sessions; *n* = 1 for 6 sessions; *n* = 1 for 7 sessions). (B) Mean percent correct performance across all participants is shown for the start (mean of first two training runs of the first session), end (mean of last two training runs of the last session) and transfer (mean of first two runs) blocks of the two sequences. (C) Mean reaction times across all participants trained on vertical vs. horizontal gratings for the start, end and transfer sessions. Error bars indicate standard error of the mean.

To test whether the observers learned the sequence structure independent of the stimuli used, we trained and tested observers with the same sequences but different stimuli. In particular, one group of participants was trained with gratings oriented left and right relative to the vertical axis and tested with gratings oriented left and right relative to the horizontal axis, while another group of participants was trained with gratings oriented left and right relative to the horizontal axis and tested with gratings oriented left and right relative to the vertical axis. Fig. 5 shows similar learning effects for both groups. Most observers (14/20) showed increased performance across training blocks, while for a small number (6/20) of weak learners (i.e. participants that reached performance less than 70% after a minimum of 4 training sessions) performance remained on average at 55.6% after training for four sessions. In particular observers’ performance improved with training and generalized fully when observers were tested with the trained stimuli but comprising of different stimuli than the trained ones. A repeated measures ANOVA on the data from all participants (including weak learners) with Session (start, end of training), Group (half of the participants were trained with gratings relative to vertical, while the rest with gratings relative to horizontal) and Sequence type (A vs. B, or C vs. D) on the data from all participants showed a significant effect of Session (*F*(1, 18) = 62.99, *p* < 0.01) but no significant effect of Group (*F*(1, 18) = 0.24, *p* = 0.629) nor a significant interaction between Session and Group (*F*(1, 18) < 1, *p* = 0.733).

We then tested whether learning of the trained sequences generalized to different stimuli. Comparing the mean of two transfer blocks to the start of training (mean of two first training blocks) showed a significant effect of Session (*F*(1, 18) = 149.82, *p* < 0.001) and no significant interaction between Session and Group (*F*(1, 18) < 1, *p* = 0.774). In contrast, comparing the mean of two transfer blocks to the end of training (mean of two last training blocks) showed no significant effect of Session (*F*(1, 18) = 3.44, *p* = 0.08) and no significant interaction between Session and Group (*F*(1, 18) < 1, *p* = 0.737). Further, performance for random sequences was on average close to chance level (55.53%). Taken together these results suggest that the observers learned the sequence structure independent of the type of stimuli presented, as learning generalized when observers were tested with the same sequence but untrained stimuli.

Analysis of reaction times (Fig. 5C) showed similar learning effects; that is observers’ responses became faster with training (*F*(1, 18) = 15.32, *p* < 0.001). This learning effect generalized to new orientations used in the transfer test. That is, there was a significant difference in reactions times between the start and transfer of training (*F*(1, 18) = 11.85, *p* < 0.01), but no significant difference was observed between the end and transfer of training to an untrained sequence (*F*(1, 18) < 1, *p* = 0.875).

### 3.4 Experiment 4

**Fig. 6:**
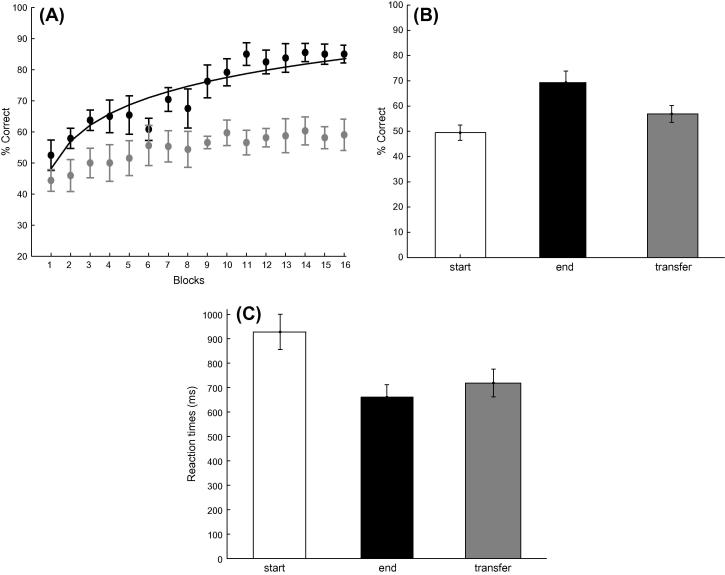
Experiment 4: (A) Behavioral performance (percent correct) across training blocks. Fitted data is shown for strong learners (black circles; *n* = 6) and weak learners (gray circles; *n* = 8). Data is shown for 16 blocks (4 training sessions); one participant completed only three sessions as performance had already saturated above 80%; while other participants required additional sessions (*n* = 9 for 5 sessions; *n* = 1 for 6 sessions). (B) Mean percent correct performance across all participants is shown for the start (mean of first two training runs of the first session), end (mean of last two training runs of the last session) and transfer (mean of first two runs) blocks. (C) Mean reaction times across all participants for the start, end and transfer sessions. Error bars indicate standard error of the mean.

To test whether attention affected the learning of sequence structure we asked observers to perform a dual task that increased attentional load. That is observers were asked to detect stimulus contrast changes as well as performing the prediction task. Fig. 6 shows that only some observers (6/14) showed increased performance across training blocks, while for several (8/14) weak learners (i.e. participants that reached performance less than 70% after a minimum of 4 training sessions) performance remained on average at 57.5% after training for four sessions. A repeated measures ANOVA with Session (start, end of training) and Sequence type (A vs. B, or C vs. D) on the data from all participants showed a main effect of Session (*F*(1, 13) = 40.58, *p* < 0.001) but no significant effect of Sequence Type (*F*(1, 13) < 1, *p* = 0.523) nor a significant interaction between Session and Sequence Type (*F*(1, 13) < 1, *p* = 0.546).

We then tested whether learning of the trained sequences generalized to untrained ones. Comparing the mean of two transfer blocks to the start of training (mean of two first training blocks) showed no significant differences in performance (*F*(1, 13) = 1.20, *p* = 0.292). In contrast, comparing the mean of two transfer blocks to the end of training (mean of two last training blocks) showed significant differences in performance (*F*(1, 13) = 21.24, *p* < 0.001). Taken together these results suggest lack of significant generalization of learning to untrained sequences. This result is consistent with Experiment 1, as observers were trained on the easier set of sequences and tested for transfer of learning on the harder set.

Analysis of reaction times (Fig. 6C) showed similar learning effects; that is, observers became faster with training (*F*(1, 13) = 10.11, *p* = 0.007). There was also a significant difference in reactions times between the start and transfer of training (*F*(1, 13) = 5.95, *p* < 0.05), but only a non-significant trend for slower reaction times was observed for the untrained sequences in the transfer test compared to the end of training (*F*(1, 13) = 2.36, *p* = 0.148). This trend became significant when the data from strong learners are considered separately from weak learners (*F*(1, 5) = 39.5; *p* = 0.001), suggesting a lack of significant learning transfer in accordance with the accuracy data.

Comparing the results of this experiment to Experiment 1 shows that when a dual task increased the attentional load fewer participants learnt the trained temporal sequence without feedback. However, we did not observe any significant differences in the magnitude of the learning effect between experiments, as shown by a non-significant main effect of Experiment (Experiment 1 vs. 4: accuracy: *F*(1, 28) < 1, *p* = 0.514; reaction times *F*(1, 28) < 1, *p* = 0.897) and a non-significant interaction between Experiment and Session (start vs. end of training) (accuracy: *F*(1, 28) < 1, *p* = 0.886; reaction times *F*(1, 28) < 1, *p* = 0.491). Only when we compared the performance of the strong learners in the two experiments, we observed significantly faster reaction times in Experiment 1 (*F*(1, 9) = 16.28, *p* < 0.01), suggesting that increased attentional load compromised performance in the prediction task.

## 4 General discussion

Learning temporal sequences is critical for developing a range of skills from language and fine motor abilities (e.g. playing a musical instrument) to navigating in a new environment. Our findings demonstrate that exposure to temporal sequences without feedback facilitates our ability to predict upcoming events. Interestingly, this improved performance lasted for a prolonged period (up to 3 months), suggesting that training resulted in knowledge of the sequence rather than simple familiarization with the task.

Successful performance in the prediction task required that the participants learned temporal order statistics across the items presented in the sequences, as the frequency of occurrence was matched for the two grating orientations. Our experimental design makes it unlikely that the participants memorized specific item positions or the two full sequences, as all stimuli were presented at the same rate and in a continuous stream, the two sequences were randomly presented across trials, the first item in the sequence was always randomized, the last three items were the same across sequences, the position of the test stimulus was randomized across trials and for half of the trials the incorrect test stimulus was presented.

Previous studies have suggested that learning of regularities may occur implicitly in a range of tasks: visuomotor sequence learning (Nissen & Bullemer, 1987), artificial grammar learning (Reber, 1967), probabilistic category learning (Knowlton, Squire, & Gluck, 1994), and contextual cue learning (Chun & Jiang, 1998). In most statistical learning studies participants are merely exposed to sequences of stimuli without being informed about the presence of statistical regularities and therefore learning is considered to occur in an incidental manner. Typically, implicit learning is inferred by poor performance in explicit recognition tasks (e.g. free recall of items present in a sequence or recognition of sequence chunks) (Curran, 1997, Kim et al., 2009, Willingham and Goedert-Eschmann, 1999). However, recent work suggests that the contribution of explicit knowledge to statistical learning may be underestimated due to the low sensitivity of the tests used to measure it (Hannula et al., 2005, Shanks and Johnstone, 1998, Shanks and Johnstone, 1999, Shanks and Stjohn, 1994). Confidence judgments have been suggested to provide a more sensitive measure of explicit knowledge; that is, high confidence ratings reflect that the participants are aware of the knowledge used to make their judgment (Bertels et al., 2012, Tunney and Shanks, 2003). In our study, we asked participants to make an explicit judgement about the identity of the upcoming test stimulus (leftward vs. rightward oriented grating) making them aware of the dependencies between the stimuli presented in the sequence. Debriefing the participants showed that participants had at least partially explicit knowledge of the sequences. That is, participants freely recalled sequence chunks, suggesting that they learned temporal order statistics among pairs or triplets of oriented gratings rather than memorizing the whole sequence. Further at the end of each session participants were asked to rate (using a scale from 1 = very unsure, to 5 = very confident) how confident they were about their responses. For the strong learners, 72% were confident (ratings of 4 or 5) that they responded correctly to the stimulus, but only 19.4% correctly guessed that they were presented with two sequences. In contrast, for the weak learners*,* only 36.8% were confident (ratings of 4 or 5) that they responded correctly to the stimulus, and only 10.5% guessed correctly that they were presented with two sequences. However, our results suggest also some potential contribution of implicit knowledge, as suggested by strong learners with lower confidence ratings.

These findings advance our understanding of the mechanisms that mediate visual learning in three main respects. First, our study is the first to test the role of sequence learning on explicit predictive judgments related to visual recognition. Previous work on learning temporal sequences has focused on implicit measures of sequence learning, such as familiarity judgments or reaction times. For example, the Serial Reaction Time Task (Nissen & Bullemer, 1987; for review see (Schwarb & Schumacher, 2012) involves participants learning visuomotor associations between spatial locations on a computer screen and response keys; locations on the screen are activated following a pre-determined sequence and participants are asked to press the corresponding keys. Training results in faster reaction times for trained than random sequences. Although such paradigms implicate that implicit learning of temporal sequences (i.e. participants are typically unable to explicitly recall the sequence or perform successfully explicit knowledge tasks) facilitates the anticipation of upcoming events, they do not test whether this knowledge can be used to predict the identity of upcoming stimuli. In contrast, we developed a new paradigm that allows us to directly test whether exposure to temporal sequences facilitates the observers’ ability to explicitly predict the identity (e.g. orientation) of the next stimulus in a sequence. Using this explicit test, we demonstrate that predictions related to the explicit recognition of objects are facilitated by previous knowledge of temporal context. Reaction time measures may reflect not only anticipation but also familiarity with the task. Our analysis of reaction times is consistent with the explicit recognition test. In particular, participants (Experiment 1) were significantly slower on untrained than trained sequences (*t*(15) = 2.56, *p* = 0.022), suggesting that the behavioral improvement we observed in the prediction task was not simply due to familiarity with the task (i.e. observers should be generally faster after training) but was specific to the trained sequence.

Second, we explored the limits of generalization afforded by statistical learning of temporal sequences. We show that performance improvement in the prediction task generalized to untrained stimulus orientations, suggesting that observers acquire knowledge of the sequence structure that is not stimulus-specific. This is consistent with artificial grammar learning studies showing positive transfer from the training stimuli to a novel set of letter strings that employ the same grammatical structure (Shanks, Johnstone, & Staggs, 1997). However, generalization to untrained sequences was limited; transfer was observed only when the untrained sequences were easier than the trained ones. When tested with easy sequences observers may identify chunks that are common between trained and untrained sequences resulting in transfer of learning to untrained sequences with a different global structure (Aslin and Newport, 2012, Koch and Hoffmann, 2000, Perruchet and Pacton, 2006).

Third, we show that attentional load compromises predictive learning in the context of temporal sequences. In particular, fewer observers were able to improve their performance in the prediction task when we employed a dual task, suggesting that attention to the temporal sequence enhances the observers’ ability to accumulate information during exposure to the sequence and predict an upcoming stimulus. Previous studies testing the role of attention in statistical learning report contradictory findings. Some studies report statistical learning during passive viewing (Fiser and Aslin, 2001, Saffran et al., 1996) or when observers perform an unrelated task (Cohen et al., 1990, Frensch et al., 1998, Saffran et al., 1997, Stadler, 1995) while others provide evidence that implicit learning (e.g. in Serial Reaction Time Tasks) is degraded under dual task conditions. (Remillard, 2003, Shanks et al., 2005). Further, selective attention between competing stimuli is shown to enhance learning of statistics related to the attended stimulus features (Baker et al., 2004, Jiang and Chun, 2001, Jiang and Leung, 2005, Jimenez and Mendez, 1999, Turk-Browne et al., 2005). Here, in accordance with previous work showing attentional modulation of sequence learning, we demonstrate that predictive learning in the context of temporal sequences is compromised but still possible (at least for some observers) when attentional load is increased by adding an orthogonal contrast task. It is possible that the effect of the dual task would be more dramatic if the orthogonal task involved the task-relevant stimulus feature (i.e. grating orientation).

In sum, our findings suggest that exposure to temporal regularities in a scene allows us to accumulate information about its structure and predict future events. In our study we used deterministic sequences but ensured that observers learned the global sequence structure rather than its items by matching the frequency of occurrence of each item in the sequence. Future work is necessary to investigate whether exposure to probabilistic temporal sequences of variable complexity, as is typically the case in natural environments, also facilitates our ability to predict future events.
